# Associations between the legal context of HIV, perceived social capital, and HIV antiretroviral adherence in North America

**DOI:** 10.1186/1471-2458-13-736

**Published:** 2013-08-08

**Authors:** J Craig Phillips, Allison Webel, Carol Dawson Rose, Inge B Corless, Kathleen M Sullivan, Joachim Voss, Dean Wantland, Kathleen Nokes, John Brion, Wei-Ti Chen, Scholastika Iipinge, Lucille Sanzero Eller, Lynda Tyer-Viola, Marta Rivero-Méndez, Patrice K Nicholas, Mallory O Johnson, Mary Maryland, Jeanne Kemppainen, Carmen J Portillo, Puangtip Chaiphibalsarisdi, Kenn M Kirksey, Elizabeth Sefcik, Paula Reid, Yvette Cuca, Emily Huang, William L Holzemer

**Affiliations:** 1 Faculty of Health Sciences, University of Ottawa School of Nursing, 451 chemin Smyth Road, Ottawa, ON K1H 8M5, Canada; 2 Frances Payne Bolton School of Nursing, Case Western Reserve University, 10900 Euclid Avenue, Cleveland, OH 44122, USA; 3 Department of Community Health Systems, University of California School of Nursing, San Francisco, CA 94143-0608, USA; 4 MGH Institute of Health Professions, CNY 36 1st Avenue, Boston, MA 02116, USA; 5 University of Hawaii School of Nursing, McCarthy Mall, Webster 439, Honolulu, HI 96822, USA; 6 University of Washington School of Nursing, Box 357266, Seattle, WA 98103, USA; 7 Office of Research & Evaluation, Rutgers College of Nursing, Ackerson Hall, 180 University Avenue, Room 330, Newark, NJ 07102, USA; 8 Hunter College, CUNY, Hunter Bellevue SON, 425 East 25 Street, Box 874, New York, NY 10010, USA; 9 Duke University School of Nursing, 20 West Bridlewood Trail, Durham, NC 27713, USA; 10 Yale University School of Nursing, PO Box 27399, West Haven, CT 06516-7399, USA; 11 University of Namibia Main Campus, Mandume Ndemufayo Avenue, Block F, Room 204, 3rd Level, Windhoek, Namibia; 12 Rutgers College of Nursing, 101 Glen Rock Road, Cedar Grove, NJ 07009, USA; 13 MGH Institute of Health Professions, 3047 Bonnebridge Way, Houston, TX 77082, USA; 14 University of Puerto Rico, PO Box 365067, San Juan, PR 00936-5067, Puerto Rico; 15 Global Health and Academic Partnerships, Brigham and Women's Hospital and MGH, Institute of Health Professions, 36 1st Avenue, Boston, MA 02129, USA; 16 University of California, 50 Beale Street, Suite 1300, San Francisco, CA 94105, USA; 17 Chicago State University College of Health Sciences, Department of Nursing, 420 S. Home Avenue, Oak Park, IL 60302, USA; 18 University of North Carolina Wilmington, 601 S. College Road, Wilmington, NC 28403, USA; 19 University of California, School of Nurisng, 2 Koret Way, San Francisco, CA 94143, USA; 20 Shinawatra University, School of Nursing, 99 Moo 10, Bangtoey, Pathumthani, Samkhok 12160, Thailand; 21 Nursing Strategic Initiatives, Lyndon B. Johnson Hospital – Executive Administration, Harris Health System, 5656 Kelley Street, Houston, TX 77026, USA; 22 Texas A&M University-Corpus Christi, 6300 Ocean Dr. Island Hall, Rm 329, Corpus Christi, TX 78404, USA; 23 The University of North Carolina at Wilmington, School of Nursing, 601 College Road, Wilmington, NC 28403-5995, USA; 24 Rutgers College of Nursing, Ackerson Hall, 180 University Avenue, Room 302C, Newark, NJ 07102, USA

**Keywords:** Ecosocial theory, HIV/AIDS, HIV-related prosecution, Jurisprudence, Sexual minority, Vulnerable populations

## Abstract

**Background:**

Human rights approaches to manage HIV and efforts to decriminalize HIV exposure/transmission globally offer hope to persons living with HIV (PLWH). However, among vulnerable populations of PLWH, substantial human rights and structural challenges (disadvantage and injustice that results from everyday practices of a well-intentioned liberal society) must be addressed. These challenges span all ecosocial context levels and in North America (Canada and the United States) can include prosecution for HIV nondisclosure and HIV exposure/transmission. Our aims were to: 1) Determine if there were associations between the social structural factor of criminalization of HIV exposure/transmission, the individual factor of perceived social capital (resources to support one’s life chances and overcome life’s challenges), and HIV antiretroviral therapy (ART) adherence among PLWH and 2) describe the nature of associations between the social structural factor of criminalization of HIV exposure/transmission, the individual factor of perceived social capital, and HIV ART adherence among PLWH.

**Methods:**

We used ecosocial theory and social epidemiology to guide our study. HIV related criminal law data were obtained from published literature. Perceived social capital and HIV ART adherence data were collected from adult PLWH. Correlation and logistic regression were used to identify and characterize observed associations.

**Results:**

Among a sample of adult PLWH (*n* = 1873), significant positive associations were observed between perceived social capital, HIV disclosure required by law, and self-reported HIV ART adherence. We observed that PLWH who have higher levels of perceived social capital and who live in areas where HIV disclosure is required by law reported better average adherence. In contrast, PLWH who live in areas where HIV transmission/exposure is a crime reported lower 30-day medication adherence. Among our North American participants, being of older age, of White or Hispanic ancestry, and having higher perceived social capital, were significant predictors of better HIV ART adherence.

**Conclusions:**

Treatment approaches offer clear advantages in controlling HIV and reducing HIV transmission at the population level. These advantages, however, will have limited benefit for adherence to treatments without also addressing the social and structural challenges that allow HIV to continue to spread among society’s most vulnerable populations.

## 

Human rights approaches to manage HIV and efforts to decriminalize HIV exposure/transmission globally offer hope to persons living with HIV (PLWH). However, among vulnerable populations of PLWH globally, substantial human rights and structural challenges still exist and must be addressed [1-4]. These challenges span all ecosocial levels of a PLWH’s context (i.e., individual, interpersonal, social and structural levels). The ecosocial structural level includes social structures (e.g., the legal system, social class) that may influence health [5-8]. In North America (Canada and the United States) the legal context of HIV may include prosecution for nondisclosure of an HIV positive serostatus to sexual partners, exposing others to HIV, and HIV transmission [9-17]. The criminalization of HIV exposure/transmission and prosecutions for HIV exposure include cases involving transmission of the virus and cases of HIV nondisclosure of an HIV positive serostatus, with or without HIV transmission. In a world that is striving to end the AIDS epidemic [18], understanding the complex challenge that prosecution of persons for HIV exposure creates for societies is essential.

Criminalization of HIV exposure/transmission is an ecosocial structural process that is embodied in “the disadvantage and injustice some people suffer… because of the everyday practices of a well-intentioned liberal society” [19,20]. In many communities, the over-use of criminal law as an HIV prevention strategy has contributed to stigma and discrimination against PLWH [14,21]. This HIV prevention strategy may be problematic in communities that have adopted the structural HIV prevention intervention of treatment as prevention and communities where test and treat programs are being scaled up [22,23]. Each of these structural HIV prevention intervention strategies are being used in North America. As indicated by UNAIDS [24] a review of existing laws that criminalize HIV exposure, HIV transmission, and nondisclosure of HIV positive serostatus can identify those laws and legal precedents that are vague or open to misinterpretation, and that lead to discrimination. However such a review is missing from the current scientific literature. The purpose of this study was to better understand the ecosocial and legal contexts in which PLWH live in North America. To that end, we examined the influence of the criminalization of HIV exposure/transmission, reported prosecutions for HIV exposure/transmission and perceived social capital on HIV ART adherence among PLWH in Canada and the United States. We used ecosocial theory and social epidemiological methods to guide our research.

## Background and significance

### HIV-related criminal law: effective policy or tool of oppression?

Structural level challenges are barriers to an individual’s HIV-related health promoting behavior and include economic, social, policy, and organizational aspects of the environmental context [25]. The criminalization of HIV exposure/transmission and the risk of prosecution based on HIV status is a controversial structural challenge faced by many PLWH globally [14,26-28]. Criminalization of HIV exposure/transmission and prosecution for HIV exposure takes on many forms including enhancements (e.g., the application of additional or more severe criminal penalties) during sentencing for other crimes when HIV is a factor in the case; court decisions that consider HIV a “deadly weapon,” resulting in PLWH being prosecuted for spitting; and laws that criminalize potential HIV transmission, without evidence of intent [14,26-28]. These prosecutions occur even when there is evidence that the PLWH has an undetectable HIV viral load and practices safer sex by using a condom, rendering HIV transmission highly unlikely [14,26-28]. Multinational and non-governmental organizations have highlighted the challenges that the criminalization of HIV exposure/transmission pose for effective HIV prevention, care, treatment, and management [26,29]. Substantial evidence suggests that the criminalization of HIV exposure/transmission has limited benefit for population health and may adversely affect individual behavior [9-11,13,14,16]. Prosecutions for HIV exposure have occurred in Canada and the United States since the beginning of the epidemic and are increasing in some jurisdictions within the two countries [12,15,27]. The current trends in prosecutions for HIV exposure and nondisclosure may create challenges for managing HIV in a way that respects human rights and dignity for all members of society. Further research is needed to guide policy-makers, courts and legislative bodies about how to interpret the scientific knowledge in an era of HIV as a manageable chronic illness, in contrast to earlier eras when HIV was perceived to be a death sentence and public health menace. In jurisdictions where prosecution for HIV exposure is possible, the extent to which prosecutions affect health promoting behaviors among PLWH is not evident and has not been studied. Understanding the effects that the threat of prosecution for HIV exposure has on an individual’s health promoting behaviors (e.g., HIV ART adherence, seeking HIV testing and treatment, serostatus disclosure) is critically important to efforts to bring about an end to the HIV epidemic.

### Social capital: collective resources and credits to achieve health

When PLWH navigate the structural challenges of their ecosocial context environment, social capital may be an essential element for the attainment of optimal health. Social capital is the “aggregate of potential resources which are linked to possession of a durable network of more or less institutionalized relationships of mutual acquaintance or recognition” [30], p.248; [31]. Social capital provides members with “credit(s)” to be used in the larger social world to achieve their interests and engage in activities and services to achieve optimal health [31-37]. In short, social capital is the resources to support one’s life chances and overcome life’s challenges [38]. Social capital defined in this way may protect PLWH from many of the structural challenges they encounter in society, because of a collective advantage. This was the case during the first decades of HIV when gay men and other minority groups rallied together to empower governments to respond to the HIV epidemics in North America and Western Europe [39,40]. The resulting work led to the development and approval of accessible HIV antiretroviral treatment and public insurance programs to cover the cost of these life-saving medications and related health care. However, mirroring global societal trends, such efforts have recently been diffuse and the social capital that was previously developed among these populations may have diminished [41].

### Racism, sexism and homophobia

The social structural phenomena of racism, sexism, and homophobia may be factors in the criminalization of HIV in some communities. These factors may be evident in the numbers of ancestral (racial/ethnic) or other minority persons who are prosecuted for HIV-related crimes or for sentencing enhancements when HIV is a factor in criminal proceedings [12,15]. The structural factors of racism and sexism have been documented as determinants of health and contribute to adverse health outcomes among PLWH globally [42,43]. Racism and sexism are forms of domination that result in oppressive power imbalances in societies [44]. Racial and sexual minorities in Canada and the United States have been disproportionately affected by the HIV epidemic and racial minorities experience higher rates of criminal prosecutions than other members of these societies. Sexism and homophobia have been attributed to the gender hierarchy in society [45]. Homophobia has been termed a weapon of sexism [46] and has been documented as a factor influencing the health and health outcomes of PLWH [47]. The criminalization of HIV and homophobia in Canada and the United States may adversely affect HIV prevention and treatment efforts among racial and sexual minority groups [9-12,14,15,26,48,49].

Currently, the global awareness about groups of persons most at risk for HIV is changing. This change in awareness is brought about by a recognition that groups who have been at risk for HIV transmission since the beginning of the epidemic remain at risk and continue to be disproportionately affected by HIV. These groups include gay and other men who have sex with men (MSM) and women of color. Although gay men in North America, Europe and Oceania were empowered in the early days of the epidemic, HIV epidemics among MSM in these regions and in most countries continue to expand [50]. HIV prevalence among MSM is substantially higher than the general male adult population globally [50]. The structural challenges in western nations that influence the health outcomes of MSM are substantially different from those in developing nations, but have profound influences on health outcomes. These structural challenges include heteronormativity and racial inequality [51,52]. In the United States, there is substantial evidence that Black MSM are more likely to be HIV-infected than white MSM [53]. This indicates the likelihood of intersecting vulnerabilities among MSM who are both sexual minorities and members of ancestral (racial/ethnic) minority groups [51]. In Canada the likelihood of acquiring HIV-infection is similar for black MSM and white MSM [53]. Legal scholars have suggested that “the gay community is an important source of values that protect against criminalization” of HIV [15]. Mutual responsibility for the prevention of HIV transmission through safer sex practices has been a hallmark of gay communities’ responses to HIV [15]. Mykhalovskiy & Betteridge [15] warn of a potential challenge for the legal context of HIV if there is erosion of the valuing of mutual responsibility for the prevention of the sexual transmission of HIV in a community with high HIV prevalence. They were concerned about the potential to overwhelm legal systems with new prosecutions for HIV nondisclosure if there is a change in valuing mutual responsibility among members of the gay community. Research is needed to inform health policy makers and guide intervention development to address the structural challenges that influence HIV-related health outcomes among gay men and other MSM.

### Theoretical framework

**Figure 1:**
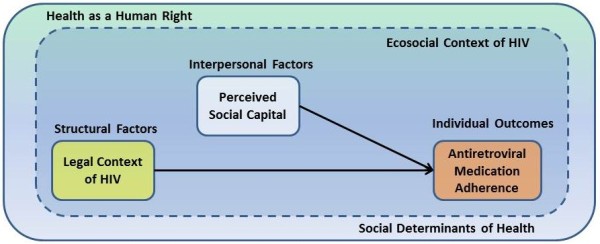
The ecosocial context of HIV-related criminal laws, social capital, and HIV antiretroviral adherence in North America.

Ecosocial theory [6] and social epidemiology methods were used to better understand the relationships between the criminalization of HIV at the structural level, individuals’ perceived social capital, and the health promoting behavior of HIV antiretroviral adherence among PLWH (Figure 1). Ecosocial theory equally values all stakeholder (e.g., PLWH, social network members, health care providers, public health and other government officials) perspectives and advocates for studying the influences of structural level policies (e.g., criminalization of HIV), both codified and enacted, across all levels of the ecosocial environment (e.g., individual, interpersonal, social and structural levels) and is mindful of the simultaneous and reciprocal effects across those levels [6,54]. Additionally, ecosocial theory is interested in exploring the pathways and power dynamics that contribute to health outcomes. The core constructs of ecosocial theory are embodiment; pathways of embodiment; cumulative interplay of exposure, susceptibility and resistance; and accountability and agency. Ecosocial theory seeks to integrate social and biological reasoning with a dynamic, historical and ecological perspective to understand population health phenomena [5-8,55]. It is compatible with the structural level concepts of health as a human right and the social determinants of health that influence health outcomes for all members of society, including PLWH [1,3,43,56]. Furthermore, ecosocial theory provides a mechanism for better understanding the intrinsic relationships that shape population health [5-8]. Krieger [7] described four intrinsic relationships to characterize populations: genealogical (relationships based on biological descent), internal and economical (relationships essential to daily activities to maintain life), external and ecological (relationships between populations and the environs they coinhabit), and teleological (conscious purpose–spanning from mutual benefit to exploitation) [7]. Social capital is shaped by genealogical relationships and encompasses internal and economical and external and ecological relationships. Building on these ecosocial theoretical foundations, we proposed that the structural process of criminalization of HIV exposure/transmission and individually perceived social capital influence ART adherence behaviors among PLWH. We considered HIV ART adherence over a 30 day period as a proxy measure for individual level health promotion engagement. This proxy measure is assumed to provide evidence of an individual’s ability to engage with the health care system (e.g., seek medical treatment), obtain necessary health care services (e.g., obtain clinic care and pharmacy services), and practice health promoting self-care behaviors (e.g., obtain prescriptions and take medications).

### Research aims

In keeping with our ecosocial theoretical framework, we had two primary research aims. Our aims were to: 1) Determine if there were associations between the social structural factor of criminalization of HIV, the individual factor of perceived social capital, and HIV ART adherence among an international sample of PLWH and 2) describe the nature of associations between the social structural factor of criminalization of HIV, the individual factor of perceived social capital, and HIV ART adherence among a North American sample of PLWH.

## Methods

The use of ecosocial theory and social epidemiology to guide our analysis provided the advantage of allowing inclusion of publically available structural level data about the legal context of HIV to be combined with our individual level survey data about HIV ART adherence, perceived social capital, and demographic characteristics among PLWH. Ecosocial theory offered contextual guidance to understand the simultaneous response across and between societal levels in environments where PLWH live [6,54]. Social epidemiological methods were used because they offer the advantage of including multiple data sources, not just traditional health and health service data to gain a contextualized understanding of what was observed [54,57].

### Ecosocial environmental data studied

This study used structural level data obtained from publically available data sources and individual level survey data obtained from the International Nursing Network for HIV Research Study V [37,58,59]. The International Nursing Network for HIV Research is an interprofessional network of researchers with extensive experience conducting clinical research with PLWH globally [60]. Study sites were selected by convenience, with principal investigators from among members of the International Nursing Network for HIV Research choosing to participate in the main study.

### Survey design and sampling strategy

This study is a sub-analysis of an international nursing collaborative study with data obtained from infectious disease clinics and AIDS service organizations from 21 sites in five countries. Survey data analyzed for this study are limited to our study sites in Canada and the United States, including Puerto Rico. The study protocol was approved by the ethics board at the coordinating site at the University of California, San Francisco (UCSF) and University of British Columbia, Behavioural Research Ethics Board; University of Washington Human Subjects Division; Northeast Ohio: University Hospitals/Case Medical Center Internal Review Board; University of Puerto Rico, Institutional Review Board; Institutional Review Board in the Texas A&M University-Corpus Christi Compliance Office; Rutgers Institutional Review Board for the Protection of Human Subjects; Institutional Review Board of the North Jersey Community Research Initiative; University of North Carolina Wilmington, Office of Research Services, Institutional Review Board; Partners Internal Review Board, Massachusetts General Hospital; Duke University Institutional Review Board; Hunter College (City University of New York [CUNY]) Human Research Protection Program (HRPP) Office; and the University of Hawaii at Manoa Committee on Human Studies. The parent study was also approved at sites in China, Namibia, and Thailand by the Shanghai Public Health Clinic Center Institution Review Board; Ethics Committee of the Ministry of Health and Social Services, Namibia; Ethics Committee of the University of Namibia; and The Ethical Committee of Lerdsin Hospital, Governmental Hospital, Bangkok, Thailand. The demographic characteristics and methods of this study have been previously published [37,58,61]. Selected demographic, survey, and environmental contextual data relevant to the aims of this study are presented.

### Study variables and data sources

Data obtained for our structural indicator, the criminalization of HIV exposure/transmission, were obtained from various publically available data sources or by conducting Internet searches to obtain the relevant information for analysis. These datasets are described in detail below. We used multiple indicators to assess the effects of the criminalization of HIV exposure/transmission on HIV ART adherence. This practice of using multiple indicators safeguards against potential problems with measurement error that can arise if a single imperfect indicator is used because measurement errors are less likely to occur if several indicators are measured [62,63].

### Legal context of HIV: criminalization and prosecutions

The legal frameworks that facilitate prosecutions for HIV exposure are rapidly changing throughout much of the world. For clarity of discussion, we use the single term laws to imply laws, regulations, and policies (including legal precedent such as the basis for HIV-related prosecutions in Canada). There are two types of laws that influence the health of PLWH globally, protective and punitive [13,16,29]. Protective laws include: (1) those that protect people living with HIV against discrimination; and (2) non-discrimination laws that specify protections for vulnerable subgroups within a population [29]. Punitive laws include: (1) HIV-specific restrictions on entry, stay or residence; (2) specifically criminalize HIV exposure or transmission; (3) criminalize same-sex sexual activities between consenting adults; (4) criminalize sex work (prostitution); (5) imposing compulsory treatment for people who use drugs and/or provide for the death penalty for drug offences [13,29]; (6) HIV-specific enhancements to sentencing for other crimes [13]; (7) criminalize exposure to or transmission of other diseases [13]; (8) requiring HIV disclosure [13]; and (9) requiring name-based HIV reporting [13]. Punitive laws present obstacles for access to prevention, treatment, care and support services for vulnerable subpopulations.

The Legal Environment for People Living with HIV (Table 1) was assessed by reviewing the relevant laws and policies in the jurisdiction (either state/province or national laws) that pertained to each study site. The types of laws, regulations, or policies were organized using eleven categories outlined by UNAIDS [29] and Lazzarini, Bray, and Burris [13]. We limited our statistical analysis to North American study sites and the following punitive legal categories: laws that specifically criminalize HIV transmission or exposure; laws that allow HIV-specific enhancements to sentencing for other crimes; laws requiring HIV disclosure; laws requiring HIV name-based reporting; and the total number of HIV prosecutions in the jurisdiction where each study site was located. We limited our analysis to these legal categories, because these legal categories were consistent across the majority of jurisdictions where study sites were located. To facilitate data analysis, we characterized the legal environment at each site by using one of three codes for each type of law, regulation or policy–“yes”, there is a law, regulation or policy; “no”, there is not a law, regulation or policy; and “no data” are available about laws, regulations or policies within the larger contextual environment where each study site was located.

**Table 1:** Legal contextual environment for people living with HIV

**Law, Policy, Regulation**	**Geographic Location of Study Site**											
	**Can**	**USA**										**PR**
		**CA**	**HI**	**IL**	**MA**	**NJ**	**NY**	**NC**	**OH**	**TX**	**WA**	
Protective												
Laws and regulations/policies that protect people living with HIV against discrimination	Yes	Yes	Yes	Yes	Yes	Yes	Yes	Yes	Yes	Yes	Yes	Yes
Non-discrimination laws or regulations that specify protections for vulnerable subpopulations	Yes	No The Americans with Disabilities Act (ADA) excludes drug users and sex workers.										
Punitive												
Laws, regulations or policies that present obstacles to access to prevention, treatment, care and support for vulnerable subpopulations	Yes	Yes, for drug users and sex workers.										
HIV-specific restrictions on entry, stay or residence	No	No*	No*	No*	No*	No*	No*	No*	No*	No*	No*	No*
Laws that specifically criminalize HIV transmission or exposure	No	Yes	No	Yes	No	Yes	No	Yes	Yes	No	Yes	No
Laws that criminalize same-sex sexual activities between consenting adults	No	No	No	No	No	No	No	No	No	No	No	No
Laws deeming commercial sex work to be illegal	No	Yes	Yes	Yes	Yes	Yes	Yes	Yes	Yes	Yes	Yes	Yes
Laws that impose compulsory treatment for people who use drugs and/or provide for death penalty for drug offences	No Data	Yes	Yes	Yes	Yes	Yes	Yes	Yes	Yes	Yes	Yes	Yes
Laws that allow HIV-specific enhancements to sentencing for other crimes	No	Yes	No	No	No	No	Yes	No	Yes	Yes	Yes	No
Laws that criminalize exposure/transmission to other diseases**	No	Yes	No	No	No	Yes	Yes	No	No	No	Yes	No
Laws requiring HIV disclosure	Yes	Yes	No	Yes	No	Yes	No	Yes	Yes	Yes	Yes	No
Laws requiring HIV reporting	Yes	No	No	No	No	Yes	No	Yes	Yes	Yes	Yes	No
Number of HIV prosecutions since 1981	13	10	0	18	4	4	4	4	25	22	8	0
Sample Size	100	300	100	95	200	100	100	200	150	228	200	100

*Note*: *CA* = California, *Can* = Canada, *HI* = Hawai’i, *IL* = Illinois, *MA* = Massachusetts, *NC* = North Carolina, *NJ* = New Jersey, *NY* = New York, *OH* = Ohio, *PR* = Puerto Rico, *TX* = Texas, *WA* = Washington, *UNK* = unknown, *United States laws that restrict entry, stay or residence based on HIV-status were repealed in January, 2010; **including communicable or contagious disease statutes that criminalize sexually transmitted infection exposure [27].

In an effort to quantify and better understand the magnitude of criminalization of HIV exposure/transmission at each of our study sites, the total number of cumulative HIV prosecutions was obtained for each jurisdiction where this information was available. HIV-related prosecutions are cumulative estimates from the beginning of the epidemic in 1981 or when records of HIV-related prosecutions became available within a geographic location. In the United States this information was obtained from the Center for HIV Law and Policy [27]*Positive Justice Project* and in Canada, this information came from the Canadian HIV/AIDS Legal Network [26] and the work of Mykhalovskiy and Betteridge [15]. Further legal contextual information for both countries was obtained from United Nations General Assembly Special Session (UNGASS) on HIV/AIDS country reports [64] and the report *Advancing HIV justice: A progress report on achievements and challenges in global advocacy against HIV criminalisation* published by the Global Network of People Living with HIV (GNP+) and the HIV Justice Network [65]. We made every effort to obtain the most accurate information on HIV-related prosecutions. However, the numbers represented here are assumed not to constitute an exhaustive representation of all HIV-related prosecutions for the jurisdictions where our study sites were located and is “likely only a sampling of a much more widespread, but generally undocumented, use of criminal laws against people living with HIV” [27] p. 201. We did not obtain information about HIV-related prosecutions or the effects of criminalization of HIV exposure/transmission from our study’s participants. Therefore, information reported here only provides contextual information that may highlight the potential challenges that HIV-related prosecution and the criminalization of HIV exposure/transmission may impose on PLWH at each study site.

### Perceived social capital

Self-reported individual-level social capital was measured using 31-items, from the 36-item Social Capital Scale [66-68]. This widely-used instrument measures eight subscales including: participation in the local community, social agency, feelings of trust and safety, neighborhood connections, friends and family connections, tolerance of diversity, value of life, and workplace connections; these items were used to create a total score. In our analysis, the three workplace connections items have been dropped, as well as two work-related questions that are part of the social agency dimension. This was due to low anticipated employment status, because employment rates among PLWH are considerably lower than the general population in developed countries [69,70]. Participants were asked to rate items on a 1–4 Likert-type scale. Higher mean scores indicate more social capital. Reliability and validity of the social capital scale have been reported as acceptable [37,66]. Cronbach’s alpha reliability for the social capital scale for our study was 0.88 and ranged from 0.84 to 0.93 for all study sites.

### HIV antiretroviral therapy adherence

For this study, HIV ART adherence was measured with a visual analogue scale for 30-day adherence [71]. This measure allows participants to self-report estimates of their percentage of adherence during this period of time. Participants were asked to mark how often they took their medications in the past 30 days on a scale of 0% of the time to 100% of the time [71]. This method is advantageous because the data reported can be directly used for analysis without calculating scores for analysis. This 30-day time frame for self-reporting medication adherence was previously validated and overcomes the challenge of remembering specific doses missed [71-73]. Further, we dichotomized our participants’ self-reported 30-day adherence and those reporting 100% adherence were categorized as adherent (coded with a value of 1) and those who adhered at a lower level (< 99%) were categorized as not adherent (coded with a value of 0). This approach exceeds the 95% adherence threshold required for sustained HIV viral suppression [74-77].

### Data analysis

All data were entered into a data management program and sent to the coordinating center (UCSF) to check the data integrity and assumptions for validity. Statistical analyses were conducted using SPSS 20. An a priori significance level of alpha = .05 was set to determine significance for all statistical tests. Logistic regression was used to assess the associations between HIV ART adherence, criminalization of HIV exposure/transmission, and perceived social capital. Measures to assess regression model constructs were derived from demographic items in the survey, the social capital scale [37,66], or data obtained from publically available data sources about laws affecting the health or social well-being of PLWH. Perceived social capital scores used the mean social capital score derived from our survey data. The indicator for HIV ART adherence (percentage adherent in the past 30-days) was used to derive a binary outcome variable (100% adherence) and to assess associations between each indicator for the legal context of HIV (e.g., prosecutions and laws), perceived social capital, and HIV ART adherence.

## Results

### Sample surveyed and selected demographic characteristics

Selected demographic characteristics for North American sites’ study participants (*n* = 1873) are summarized in Table 2. Participants were primarily male (*n* = 1299; 69.4%) with an average age of approximately 46 years (range 18–74 years). Most participants reported less than adequate income (*n* = 1940; 89%), although 20% (*n* = 367) reported that they were employed. Most participants (*n* = 1500; 82%) were prescribed and currently took HIV ART. More than half of the participants (*n* = 1030; 58.1%) self-reported an undetectable HIV viral load. The majority of participants attributed their acquisition of HIV to having sex with a man (*n* = 1239; 73%). Of these, 64% (*n* = 791) were male, 32% (*n* = 396) were female, and the remainder were transgender, other, or declined to report their gender identity.

**Table 2:** Selected demographic and HIV disease characteristics (n = 1873)

	**Frequency (%)**		**Mean (SD)**
Age (years)			46.1 (± 9.2)
Gender			
Male	1299 (69.4)		
Female	503 (26.9)		
Transgender/Other	51 (2.8)		
Ancestry (Race/Ethnicity)			
African Am/Black	755 (40.3)		
Latina/Latino	425 (22.7)		
White	488 (26.1)		
Other	179 (9.7)		
Education			
11^th^ grade or less	491 (26.2)		
High School	735 (39.2)		
2+ yrs College	630 (33.9)		
Income Adequate	495 (21.1)		
HIV Disease Indicators			
Year diagnosed with HIV			2,000 (± 6.6)
Prescribed HIV antiretroviral therapy	1500 (82.3)		
Has AIDS diagnosis	788 (42.1)		
Undetectable Viral Load	1030 (58.1)		
	Frequency (%)		
HIV transmission method^a^	Man	Woman	Transgender/Other
Sex HIV+ man	791 (67.5)	396 (83.9)	42 (89.4)
Sex HIV+ woman	349 (33.3)	19 (4.8)	11 (36.7)
Sharing needles	301 (28.3)	122 (28.8)	17 (50)
Blood transfusion	76 (7.6)	41 (10.2)	6 (20.7)
Don’t know	124 (12.8)	34 (8.7)	4 (17.4)

Note. ^a^ Participants were asked to list the possible ways they could have been infected with HIV. Responses were not mutually exclusive and participants may have listed multiple modes of transmission. Transgender/Other includes persons identifying as a transman, transwoman, genderqueer, other, or decline to state.

### Legal context of HIV: criminalization and prosecutions

The legal context of HIV includes the laws, regulations, policies, and legal precedents that influence the lives of PLWH. The laws that contribute to HIV-related prosecutions and the numbers of persons being prosecuted for HIV-related offenses vary widely across our study sites (Table 1). Canada has no criminal legislation related to HIV transmission or disclosure, but legal precedent and Supreme Court of Canada decisions since 1998 have resulted in more than 114 HIV-related prosecutions [15,78]. The majority (approximately 69%) of HIV-related prosecutions in Canada occurred after 2003 [15]. Many states in the United States have HIV-specific criminal legislation (e.g., California, Illinois, New Jersey, North Carolina, Ohio, and Washington). In the United States, states and territories without HIV-specific laws, regulations or policies may have established legal precedent or other policy mechanisms through which PLWH can be prosecuted or have their sentences enhanced based on HIV status. For example, New York law does not define whether criminal penalties apply to HIV exposure, however courts there in at least two cases have considered HIV a “deadly weapon” in assault cases [27].

Among jurisdictions where our study sites were located, British Columbia has had 13 HIV-related prosecutions and Canada has had the highest per capita rate of HIV prosecutions among the two countries where our study sites are located (*n* = 114) [15,28]. Additionally, these prosecutions have increased dramatically since 2003 [15]. For comparison, in the United States there have been 350 prosecutions for HIV-related crimes [27]. Among our study sites in the United States, Hawaii and Puerto Rico currently have no punitive legal policies toward PLWH and there have been no reported HIV-related prosecutions in these jurisdictions. Among our study sites, the states with the most HIV-related prosecutions were Ohio (*n* = 25) and Texas (*n* = 22). Three jurisdictions where our study sites were located, Canada, Illinois, and Ohio, are among the world’s top 30 jurisdictions for HIV criminalization [65].

### Perceived social capital

Among our North American study sites, we observed moderately high levels of mean social capital scores ranging from 2.53 ± 0.56 in California to 2.79 ± 0.55 in North Carolina (possible scale range 1–4, higher values signify greater perceived social capital). On average, participants reported a mean social capital score of 2.63 ± 0.55. The mean social capital score for our Canadian (2.54 ± 0.56) and California sites (2.53 ± 0.59) were low in comparison to other North American sites. Perceived mean social capital scores were highest in Hawai’i (2.73 ± 0.56), Puerto Rico (2.74 ± 0.52), and North Carolina (2.79 ± 0.55; Table 3).

**Table 3:** Social capital and HIV-antiretroviral adherence among people living with HIV

**Site**	***N***	**Social Capital *μ *(sd)**	**Adherence % past 30-day *μ *(sd)**	**Adherence 100%**^**a **^***n *(%)**	**Prescribed HIV antiretroviral therapy *n *(%)**
Canada	100	2.54 (0.56)	84.1 (24.7)	37 (43.5)	85 (85)
Puerto Rico	100	2.74 (0.52)	88.7 (20.1)	51 (56)	91 (91)
California	300	2.53 (0.59)	82.7 (23.2)	86 (36.8)	234 (78)
Hawaii	100	2.73 (0.56)	89.6 (14)	32 (35.2)	91(91)
Illinois	95	2.66 (0.59)	83.4 (24.4)	29 (36.3)	80 (84)
Massachusetts	200	2.67 (0.54)	84.8 (23.2)	70 (40.7)	172 (86)
New Jersey	100	2.64 (0.48)	85.1 (21.9)	36 (43.4)	83 (83)
New York	100	2.69 (0.59)	83 (23.7)	31 (37.8)	82 (82)
North Carolina	200	2.79 (0.55)	86.1 (19.7)	78 (43.3)	180 (90)
Ohio	150	2.59 (0.52)	86.1 (20.9)	53 (41.4)	128 (85)
Texas	228	2.64 (0.52)	89.5 (19)	109 (55.6)	196 (86)
Washington	200	2.54 (0.56)	85.8 (23.8)	87 (51.8)	168 (84)
Total	1,873	2.63 (0.55)	85.7 (21.8)	699 (44)	1,590 (84.9)

*Note*. ^a^ = computed from 30-day adherence data; possible mean social capital scores range from 1 indicating poor social capital to 4 indicating high social capital.

### HIV antiretroviral therapy adherence

Among our participants, the number of participants who reported being prescribed HIV ART was high at 84.9% (n = 1,590), range 78% in California to 91% in Hawai’i and Puerto Rico. Self-reported HIV antiretroviral adherence was fair with mean 30-day adherence reported at 85.7% of prescribed doses. Median 30-day adherence was 95%. Fully, 52% (*n* = 827) of our participants were categorized as more than 95% adherent. The level of adherence was highest among participants in Texas (mean 30-day adherence, 89.5%; 60% [*n* = 118] greater than 95% adherent) and lowest among participants in California (mean 30-day adherence, 82.7%; 45.7% [*n* = 107] greater than 95% adherent) and New York (mean 30-day adherence, 83%; 39% [*n* = 32] greater than 95% adherent). The largest percentage of participants who reported greater than 95% adherence was in Washington 60.7% (n = 102; Table 3).

### Correlation and logistic regression analyses

To determine if there were associations between the social structural factor of criminalization of HIV exposure/transmission, perceived social capital, and HIV ART adherence among an international sample of people living with HIV, a correlation was computed (Table 4). Spearman’s rho statistic was used to determine correlations because two variables were skewed, past 30-day adherence (skewness = −2.14) and number of HIV prosecutions (skewness = 3.11). Mean perceived social capital score, *r* (1588) = .168, *p* < .01; HIV disclosure required by law, *r* (1588) = .065, *p* = .01; and HIV exposure/transmission is a crime, *r* (1588) = −.052, *p* = .04 were all significantly associated with 30-day adherence. The positive association (*r* [1588] = .168, *p* < .01) between mean social capital score and 30-day adherence means that PLWH who reported higher perceived social capital also reported better average 30-day adherence. The positive association (*r* [1588] = .065, *p* = .01) between HIV disclosure being required by law and 30-day adherence means that PLWH who live in areas where HIV disclosure is required by law reported better average 30-day adherence. PLWH who live in areas where HIV exposure/transmission is a crime reported lower 30-day adherence as evidenced by a negative association (*r* [1588] = −.052, *p* = .04; Table 4). Although significantly associated, each of these three variables had a weak association with 30-day adherence. Using *r*^*2*^ as an indicator of effect size, 3% of the variance observed in 30-day adherence can be explained by perceived social capital. The variables HIV disclosure is required by law and HIV exposure/transmission is a crime each explained less than 1% of the variance observed in 30-day adherence.

**Table 4:** Associations between criminalization of HIV, perceived social capital, and HIV antiretroviral adherence

**Variable**	**Correlations**							
	30-day adherence	100% adherent^a^	Perceived Social Capital	HIV Prosecutions	HIV Exposure/Transmission Law	HIV Sentencing Enhanced	Other Disease Exposure/Transmission Law	HIV Disclosure Law
100% adherent^a^	.901**							
Perceived Social Capital	.168**	.125**						
HIV Prosecutions	.006	.015	-.098**					
HIV Exposure/Transmission Law	-.052*	-.038	-.050*	-.161**				
HIV Sentencing Enhanced	.008	.027	-.099**	.521**	.225**			
Other Disease Exposure/Transmission Law	-.049	-.025	-.086**	-.064**	.465**	.518**		
HIV Disclosure Law	.065**	.078*	-.009	.394**	.225**	.144**	-.145**	
HIV Reporting Law	.004	.022	-.072**	.680**	.678**	.389**	.217**	.631**

*Note*: ^a^ = computed from 30-day adherence data, ** *p* < .01 (2-tailed), *p < .05 (2-tailed).

Logistic regression was used to estimate the probability of being 100% adherent in our sample of North American adults living with HIV. Predictor variables were entered into the statistical model based on our ecosocial theoretical framework. Consistent with this framework, in the first block, we entered the individual level demographic variables, gender, age, ancestry (race/ethnicity), education, and years since HIV diagnosis. Next, the social network resources variable of perceived social capital was added. In the final block we added the HIV legal context variables, number of HIV-related prosecutions, laws that specifically criminalize HIV exposure or transmission, laws that allow HIV-specific enhancements to sentencing for other crimes, laws that criminalize exposure/transmission to other diseases, laws requiring HIV disclosure, and laws requiring HIV reporting. As shown in Table 5, individual level demographic characteristics (block 1 likelihood ratio chi-square = 44.44 [9]*p* < .001) and social network resources (block 2 likelihood ratio chi-square = 25.35 [1]*p* < .001) contributed statistically significantly to the overall model. The demographic characteristics that contributed the most to adherence were age and ancestry (race/ethnicity). Older participants were more likely to be adherent than younger participants. Participants whose ancestry was Hispanic (Latino[a]) or White/Anglo were more likely to be adherent than participants from any other ancestry category. Hispanics were twice as likely to be adherent and Whites were 2.4 times more likely to be adherent. The HIV legal context variables entered in Block 3 did not statistically influence the final model significantly (block likelihood ratio chi-square = 10.66 [6]*p* = .093). However, the legal context variable of HIV disclosure law did predict HIV ART adherence among our participants. Persons in our sample who lived in jurisdictions where HIV disclosure was required by law were 1.4 times more likely to be adherent than those in areas where HIV disclosure was not required by law (*p* =.054). The logistic regression model successfully predicted HIV ART adherence with the overall model and 4 predictors (i.e., age, ancestry [race/ethnicity], perceived social capital, and HIV disclosure law) achieving statistical significance and classification results indicated modest success, with an overall correct classification of 60%. The overall effect size for the full model was also modest, with Nagelkerke’s *R*^*2*^ equal to .072 [79,80]. Sensitivity analyses using lesser degrees of HIV ART adherence (e.g., 80-95%) were not statistically significant.

**Table 5:** Logistic regression summary for variables associated with HIV antiretroviral adherence (n = 1455)

				**95% CI, Odds ratio**	
**Predictor**	**B (SE)**	**Wald**	**Odds Ratio**	**Lower**	**Upper**
Block 1: *Individual Level Demographic Characteristics* (*X*^*2*^ = 44.44, *df* = 9, *p* < .001)					
Gender	-.156 (.096)	2.642	.855	.708	1.033
Age	.016 (.007)	5.453*	1.016	1.003	1.030
Ancestry^a^		30.577**			
Asian/Pacific Islander (*n* = 39)	.055 (.472)	.013	1.056	.419	2.663
African American/black (*n* = 581)	.153 (.335)	.207	1.165	.604	2.248
Hispanic/Latino(a) (*n* = 343)	.722 (.349)	4.270*	2.058	1.038	4.080
Native American Indian (*n* = 47)	.060 (.460)	.017	1.061	.431	2.616
White/anglo (non-Hispanic) (n = 398)	.859 (.337)	6.498*	2.361	1.220	4.570
Education	-.033 (.054)	.364	.968	.870	1.077
Year diagnosed with HIV	.011 (.008)	1.750	1.011	.995	1.027
Block 2: *Social Network Resources* (*X*^*2*^ = 25.35, *df* = 1, *p* < .001)					
Perceived Social Capital	.517 (.104)	24.834**	1.676	1.368	2.054
Block 3: *HIV Legal Context* (*X*^*2*^ = 10.66, *df* = 6, *p* = .093)					
HIV Prosecutions	-.003 (.004)	.418	.997	.989	1.006
HIV Exposure/Transmission Law	-.176 (.257)	.469	.838	.506	1.388
HIV Sentencing Enhanced	-.005 (.149)	.001	.995	.742	1.334
Other Disease Exposure/Transmission Law	.118 (.160)	.543	1.125	.823	1.538
HIV Disclosure Law	.321 (.166)	3.726*	1.379	.995	1.911
HIV Reporting Law	.200 (.322)	.385	1.221	.650	2.295
Constant, overall model	−24.500 (16.569)	2.186			

Note: Model *X*^*2*^ = 80.66, *df* = 16, *p* < .001); Nagelkerke *R*^*2*^ = .072; percent correctly classified = 60%; pmeter values reported are from the final logistic regression model; ^a^reference category for ancestry is other (n = 47); * *p* ≤ .05; ** = *p* < .01.

## Discussion

The core constructs and intrinsic relationships of ecosocial theory provide a means to better understand structural factors that influence HIV antiretroviral adherence, including criminalization of HIV exposure/transmission. Embodiment is a multilevel phenomenon that integrates soma, psyche, and society, within historical and ecological context [6] occurring through genealogical relationships that provides a clue to life histories, both hidden and revealed [6]. Genealogical relationships also shape pathways to embodiment in the ecosocial environment that involve “exposure, susceptibility, and resistance (as both social and biological phenomena), structured simultaneously by societal arrangements of power… and constraint’s and possibilities of our biology” [6], p. 225. Internal and economical, and external and ecological intrinsic relationships shape accountability and agency (who and what is responsible for social inequities in health and for rectifying them) [6] among members of society. From an ecosocial theory perspective, it is important to consider the influence of HIV criminal laws and social capital on HIV antiretroviral adherence from multiple levels (e.g., individual, neighborhood, political jurisdiction, national) and in multiple domains (e.g., home, work, other public settings). This study’s participants represent many of the most vulnerable members of North American society.

### HIV antiretroviral therapy adherence

Our analysis of the ecosocial factors influencing HIV ART adherence in North America identified significant positive associations between perceived social capital, HIV disclosure required by law, and self-reported HIV ART adherence. However, it is worth noting that there is no gold standard of HIV ART adherence measurement. All measures of HIV ART adherence have limitations [71,74,81,82]. Medication event monitoring system (MEMS) use is difficult to implement because of high cost and patient resistance/lack of cooperation and difficulty to monitor MEMS devices and data retrieval when study visits are as long as 6 months apart. Pill counts, pharmacy refill data, and therapeutic plasma drug monitoring all have drawbacks that limit their utility. Considering these factors and the inter-correlations among these measurement approaches [82,83], we used two well-validated adherence measures. The visual analog scale (VAS) developed by Walsh [71,84] that assesses 30-day adherence reporting septely for each drug along a continuum anchored by “none of my doses” to “every one of my doses.” This measure has shown to be correlated with other measures of adherence, such as MEMS [71,85] and a 30-day timeframe has recently been supported as preferable to other approaches of self-report [73]. We also used a 30-day adherence rating scale that has been recommended by Lu and colleagues [73], in which participants are asked “Thinking back over the past 30 days, rate your ability to take all your medications as prescribed” (6 response ratings: Very poor, Poor, Fair, Good, Very good, and Excellent). This approach has yielded the least over-reporting when compared to MEMS. These approaches, however, are still susceptible to reporting biases and likely suggest an overestimate of actual adherence. Conservatively, we chose to dichotomize at perfect vs. less than perfect adherence; finer gradations in the self-reported adherence data (e.g., 90%, 80%) are questionable given the nature of the instrument and the imprecision of recall and reporting of medication-taking behavior.

Our statistical analyses were only sensitive to the most stringent rates of HIV ART adherence (i.e. 100% adherence). This finding leads us to believe that longitudinal research that explore our model constructs is needed to more fully understand how HIV-related criminal prosecutions and perceived social capital influence HIV ART adherence among persons living with HIV in North America. Future studies should include both longitudinal and multi-site study designs that specifically inquire about PLWH’s encounters with the criminal justice system in addition to measuring their perceptions of social capital and HIV ART adherence. In addition to quantitative studies, there needs to be qualitative research that explores at the individual level the influence that HIV-related criminal law approaches have on the lives of PLWHs and how they navigate their experiences with stigma and discrimination that these laws perpetuate.

Many factors that influence a person’s HIV antiretroviral adherence have been documented, including individual factors (e.g., anxiety, depression, illicit drug use, knowledge about disease and medication regimen), interpersonal factors (e.g., social support, effective patient-provider relationships), and social/structural factors (e.g., homelessness, access to care and treatment). Among our study’s participants, evidence of the influence of anxiety and depression on HIV antiretroviral adherence has been documented [86]. Support for adherence self-efficacy predicted adherence behavior, among our participants and may partially mediate environmental influences and cognitive or personal factors [59]. Additionally, factors that mediate HIV antiretroviral adherence among participants include sense of coherence (“overall well-being and ability to cope with stress” [86]), self-compassion, and engagement in care [58]. The information from these studies highlights the effects of individual and interpersonal level factors that influence adherence among our participants and suggest that additional structural factors may also influence adherence. The criminalization of HIV exposure/transmission may not be the only structural level factor that influences adherence among our study participants. Homelessness and access to care and treatment may also influence adherence among our study’s participants, however we do not have data to support these conclusions.

### Criminalization of HIV exposure/transmission and HIV-related prosecutions

HIV antiretroviral adherence is an essential component of managing HIV at both individual and population levels. Our study’s use of ecosocial theory, allows us to better understand the internal and economical and external and ecological intrinsic relationships and the cumulative interplay among exposure, susceptibility, and resistance that influence HIV antiretroviral adherence as part of the embodied social ecology for our study’s participants. Our findings related to adherence among PLWH who live in jurisdictions were the potential for HIV-related prosecutions exist underscore the complex social dynamics at play in the discourse about the use of criminal law to manage HIV disease. The challenge of balancing individual rights to freedom of sexual expression and the protection of the health of a population are at odds here. Criminalization of HIV exposure/transmission as a prevention intervention has limited efficacy [9-11,14,17] and does not provide sufficient protection for persons who engage in high risk sexual behavior, who may assume they are protected because there is a law to protect them. The continued practice of prosecuting persons for HIV-related “crimes” may actually limit the effectiveness of other HIV prevention interventions. There is also evidence that continued HIV-related prosecutions may reduce the likelihood that persons who know their HIV status will seek treatment [87]. Human rights based HIV prevention interventions may allow us to capitalize on altruistic behaviors exhibited by PLWH [14,17].

The teleological intrinsic relationship described by Krieger [7] and our findings of associations between the legal context of HIV and ART adherence may be evidence of the existence of the concept of “therapeutic citizenship” in North America. This concept has been used to characterize rates of adherence to ART among PLWH in developing nations [88]. The concept is based on the view that citizenship is “enacted through a web of institutional and political cultures, rather than the more classic understanding of the political relationship between citizen and state” [88] p. 34. Nguyen et al. [88] used the concept of therapeutic citizenship to understand exemplary adherence observed in societies whose governments have severely limited economic resources for the provision of health care and social services. In these societies, PLWH are challenged with retaining familial and social networks while navigating and accessing health care and social service resources from governmental agencies. They described environments where PLWH appropriate ART as a set of rights and responsibilities that facilitate their negotiation of conflicting moral economies in their fight to manage HIV and remain viable members of society [88].

We contend that the concept of therapeutic citizenship among PLWH is applicable in Canada and the United States because the potential for prosecution for HIV nondisclosure, exposure, or transmission creates conflict between the individual and society. Our findings of higher rates of adherence in geographic locations where prosecutions for HIV occur may be partially explained by a desire among PLWH to be “good therapeutic citizens” and protect others from exposure to HIV [14]. An alternate explanation for this finding may be that PLWH fear being accused of not being a good therapeutic citizen and facing the potential for HIV-related prosecution [17]. Through the practice of adherence to HIV antiretroviral medications, PLWH reduce the likelihood of exposing others to HIV. We suspect that our observation of increased adherence to HIV ART in jurisdictions where HIV disclosure is a legal requirement is partially explained by “therapeutic citizenship” being enacted by our participants and an example of their ethical commitment to protect others from exposure to HIV [14].

From a public health perspective, it is disconcerting that HIV-related prosecutions and the criminalization of HIV exposure/transmission continues. HIV is currently the only disease for which people can be prosecuted even if they do not transmit the virus to another person. The criminalization of HIV exposure/transmission as a structural HIV prevention intervention may create a “catch 22” scenario for PLWH. PLWH who work to achieve optimal health and engage in practices to be a good therapeutic citizen by adhering to HIV ART may lose hope for living a normal life in a society that does not accept them as human beings. The continued practice of HIV-related prosecutions and criminalization of HIV exposure/transmission contributes to the creation of a viral underclass [89] that faces stigma and discrimination with the threat of prosecution and incarceration [17].

### Perceived social capital

The intrinsic relationships within ecosocial theory most relevant to understanding perceived social capital in our study are internal and economical and external and ecological relationships. Our findings related to perceived social capital highlight the importance of social networks and the collective ability of PLWH to navigate the challenges they face in their daily lives. Perhaps this is because our study participants hold similar socioeconomic position within their respective geographic locations. This leads us to believe that the importance of social capital and the “credits” it offers individual members of a society may be essential elements in the global fight to end the HIV epidemic. In addition, our findings related to perceived social capital reinforce the importance of the concept of therapeutic citizenship as a collective benefit to PLWH and all members of societies where they live.

### Limitations

Our study was influenced by an overrepresentation of factors that are relevant to contexts within the United States because of the large number of surveys collected within the United States. This limitation reduces the ability to generalize findings outside the United States, because there may be insufficient power to determine statistical differences for our Canadian site. The non-random recruitment strategy used may introduce bias, which may be especially evident in the level of perceived social capital, because persons participating may have sufficient social capital to gain access to the health care resources they need, including participation in research studies. Our reliance on ART adherence self-report survey data and lack of biological markers to assess adherence may have resulted in biased data. In the absence of an affordable and non-invasive adherence measure [90], we believe that reliance on the 30-day self-report visual analogue scale used provided valid and sufficient ART adherence information to determine the effects of structural factors influencing ART adherence. Additionally, our study’s survey responses may be influenced by social desirability bias.

Additional limitations indicative of the complexity of studying structural challenges influencing HIV include the challenge of obtaining accurate and current legal and policy information related to the criminalization of HIV and how ancestry (race/ethnic) data were collected. Our use of the United States’ Census Bureau ancestry (race/ethnic) categories complicates interpretation of data from our Canadian study site [51]. Use of this classification system may be insufficient to accurately represent epidemic trends in Canada and the United States. Our study findings provide limited evidence about the effects of HIV-related criminal laws and social capital on HIV ART adherence among women, transgender, and other minority groups. Thereby limiting our ability to understand the effect of HIV-related criminal laws on the HIV ART adherence behaviors of women, transgender, and other minority members of society.

## Conclusions

In this study, we observed weak but significant associations between the structural factor of criminalization of HIV exposure/transmission and its association with HIV ART adherence. This observation was associated with the individuals’ perceived social capital. Our finding of associations between HIV ART adherence and indicators from the structural level of criminalization of HIV exposure/transmission and the individual’s perceived social capital provide evidence to help better understand the ecosocial context of HIV and its’ influence on health promoting behavior among PLWH. These findings highlight the importance of addressing not only biomedical issues, but the social and structural challenges that influence health behavior among PLWH. In both our correlation and regression analyses, perceived social capital consistently emerged as an indicator associated with HIV ART adherence. This observation underscores the importance of considering social capital in the effective management of HIV disease globally. Current biomedical approaches to HIV offer the technological advantage of controlling HIV at the molecular and cellular levels [91]. These advantages however, will have limited benefit without also addressing the social and structural challenges that allow HIV to continue to spread among societies’ most vulnerable and at risk populations. Overcoming these challenges requires intersectoral cooperation and the political will to implement evidence-informed health policies and legal reforms aimed at achieving optimal health and wellness for all.

## Abbreviations

AIDS: Acquired immune deficiency syndrome; ART: Antiretroviral therapy; HIV: Human immunodeficiency virus; mL: Milliliter; MSM: Men who have sex with men; PLWH: Person living with HIV; UNAIDS: Joint United Nations Programme on HIV/AIDS.

## Competing interests

The authors declare that they have no competing interests.

## Authors’ contributions

This work was completed as part of an international research collaborative, and was only possible because of each site investigator’s hard work and commitment to the project. Each author substantially contributed to designing the research protocol, collected data, assisted in the analysis, and in the writing of this manuscript. Therefore, each author made significant contributions to this work as outlined below and we want to acknowledge these contributions. Each author has approved the final version of this manuscript. JCP conceived the research question and design, collected data, completed the analysis, and interpreted of data, and wrote the first draft of the manuscript. ARW contributed to developing the research question, study design, collected data assisted with the theoretical model development, assisted with the analysis and interpretation of the data and assisted Dr. Phillips in writing the manuscript. CDR, contributed to developing the research study design, collected data, assisted with the analysis and assisted Dr. Phillips in writing the manuscript. IBC contributed to developing the research study design, collected data and assisted with the interpretation of the data and assisted Dr. Phillips in writing the manuscript. KMS contributed to developing the research study design, collected data, assisted with the theoretical model development, analysis and interpretation of the data and assisted Dr. Phillips in writing the manuscript. JV contributed to developing the research study design, collected data, assisted with the theoretical model development, and assisted with the interpretation of the data and assisted Dr. Phillips in writing the manuscript. DW contributed to developing the research study design, collected data and assisted with the analysis and interpretation of the data and assisted Dr. Phillips in writing the manuscript. KN contributed to developing the research study design, translated the instruments and protocol, collected data and assisted with the interpretation of the data and assisted with the analysis and interpretation of the data and she assisted Dr. Phillips in writing the manuscript. JB contributed to developing the research study design, collected data and assisted with the analysis and interpretation of the data and he assisted Dr. Phillips in writing the manuscript. WTC contributed to developing the research study design, translated the instruments and protocol, collected data and assisted with the analysis and interpretation of the data and she assisted Dr. Phillips in writing the manuscript. SI contributed to developing the research study design, translated the instruments and protocol, collected data and assisted with the analysis and interpretation of the data and assisted Dr. Phillips in writing the manuscript. LSE contributed to developing the research study design, collected data and assisted with the interpretation of the data and assisted Dr. Phillips in writing the manuscript. LTV contributed to developing the research study design, collected data and assisted with the interpretation of the data and assisted Dr. Phillips in writing the manuscript. MRM contributed to developing the research study design, collected data and assisted with the interpretation of the data and assisted Dr. Phillips in writing the manuscript. PN contributed to developing the research study design, collected data and assisted with the interpretation of the data and assisted Dr. Phillips in writing the manuscript. MOJ contributed to developing the research study design, collected data and assisted with the analysis and interpretation of the data and he assisted Dr. Phillips in writing the manuscript. MM contributed to developing the research study design, translated the instruments and protocol, collected data and assisted with the analysis and interpretation of the data and she assisted Dr. Phillips in writing the manuscript. JK, contributed to developing the research study design, collected data and assisted with the interpretation of the data and assisted Dr. Phillips in writing the manuscript. CJP, contributed to developing the research study design, translated the instruments and protocol, collected data and assisted Dr. Phillips in writing the manuscript. PC contributed to developing the research study design, translated the instruments and protocol, collected data and assisted with the analysis and interpretation of the data and assisted Dr. Phillips in writing the manuscript. KMK contributed to developing the research study design, collected data and assisted with the interpretation of the data and assisted Dr. Phillips in writing the manuscript. ES contributed to developing the research study design, collected data and assisted with the interpretation of the data and assisted Dr. Phillips in writing the manuscript. PR contributed to developing the research study design, collected data and assisted with the analysis and interpretation of the data and she assisted Dr. Phillips in writing the manuscript. YC managed the study database and coordinated communication between all study sites and assisted Dr. Phillips in writing the manuscript. EH managed the study database. WLH contributed to developing the research study design, collected data and assisted with the analysis and interpretation of the data, and he assisted Dr. Phillips in writing the manuscript. Each author has read and agrees with the final version of the manuscript submitted to BMC Public Health.

## Pre-publication history

The pre-publication history for this paper can be accessed here:

http://www.biomedcentral.com/1471-2458/13/736/prepub
